# Threats to the emotional wellbeing of mainland Chinese students studying in Australia: an interpretivist study

**DOI:** 10.1080/17482631.2023.2221912

**Published:** 2023-06-13

**Authors:** Jian Zhao, Elaine Chapman, Tom O’Donoghue

**Affiliations:** The University of Western Australia (M098), 35 Stirling Highway, Perth Western, Australia

**Keywords:** Mainland Chinese postgraduate students, international students, Australian higher education, mental health, wellbeing

## Abstract

**Purpose:**

In 2017, international students contributed almost $32 billion to Australia’s economy, more than half of which was attributable to students from China. Despite its historical popularity as a study destination, research suggests that these students confront numerous obstacles in pursuing their studies within Australia. In this study, the perspectives of these students were explored. The dominant issues raised by these students related to mental health and emotional wellbeing.

**Methods:**

Nineteen students in one Australian university participated in one-on-one in-depth semi-structured interviews. Data were analysed using grounded theory approaches. Three broad themes were generated in the study: psychological stress (which was linked to language barriers, shifts in pedagogy, and changes in lifestyle); perceived safety (which was linked to lack of security, safety and perceived racial discrimination); and social isolation (linked to reduced sense of belonging; lacking close personal connections; and feelings of loneliness and homesickness).

**Conclusions:**

Results suggested that a tripartite model of interactive risk factors may be appropriate for exploring how international students fare emotionally with their new environments.

## Introduction

International students make a significant contribution both to Australia’s economy and to its cultural diversity (C. C. Wang et al., 2015; McGowan, 2018; The Australian Government, 2016). Mainland Chinese students accounted for 38.03% of all international students enrolled in Australian universities in 2019 (The Australian Government, 2019), a significant proportion of whom arrived to do Master’s and Doctoral level degrees. Various studies have, however, highlighted the challenges that students at this level can confront whilst studying overseas (Busher et al., 2015; Dawborn-Gundlach, 2015, 2018). Given the critical role that international students will play in Australia’s recovery from the COVID-19 pandemic, it is critical that research be conducted on how students can be assisted to address these challenges (Zhao, Chapman, et al., 2022; Zhao, Houghton, et al., 2022).

Numerous studies have been conducted on international students’ experiences in Australia (L. T. Tran, 2011; Mckenzie & Baldassar, 2017; Yue & Lê, 2013), including those of Chinese International Students (Anderson & Guan, 2018; Chen, 2008; C. L. L. T. Tran, 2014; Lu et al., 2014; Pan & Wong, 2011; Redfern, 2016; Zheng et al., 2004). These studies have, however, typically been conducted within the positivist paradigm, in which pre-defined theoretical constructs such as that of “adjustment” have been explored via survey questionnaires of undergraduate students (Anderson & Guan, 2018; Chen, 2008; Lu et al., 2014; Pan & Wong, 2011; Q. Wang et al., 2011; Zheng et al., 2004).

International students pursuing Master’s or Doctoral degrees in Australia may face unique challenges in the course of their studies, relative to undergraduate students. These challenges can arise from various aspects of academic, cultural, and social life. For example, the academic challenges confronted by postgraduate students are likely to be more complex than those confronted by other students, as they will be typically be undertaking more advanced, specialized coursework units, as well embarking on their own independent research (Karhbet, 2015). Concurrently, postgraduate students often receive less structured support from peers and faculty in comparison to undergraduate students. This may lead to feelings of isolation and increased stress, particularly for those who are new to the Australian academic environment (Karhbet, 2015). Further still, many postgraduate students will be mature-age students, who are more likely than immediate school-leavers to have family responsibilities that must be met in addition to the demands of their studies. Such obligations can affect these students’ engagement in on-campus activities, and have a further negative impact on their sense of belonging to the university (Fambely, 2020; Karhbet, 2015; Stachl & Stachl et al., 2020).

The present paper arose from a larger project on the perspectives of Chinese international students on their studies in Australian universities. While the study was conducted to explore the participants’ experiences while pursuing postgraduate study in Australia in a relatively broad sense, the dominant issues raised by them pertained to their mental health and emotional wellbeing. The aim of the subsequent focused study reported here was to generate theory on perspectives in that regard.

The World Health Organization (17 June 2022) defined mental health as “a state of mental well-being that enables people to cope with the stresses of life, realize their abilities, learn well and work well, and contribute to their community” (para. 1). Citing Keyes (2003), Langeland (2014) defined emotional wellbeing as “a positive balance of pleasant to unpleasant affect and a cognitive appraisal of satisfaction with life in general” (p.1874). These definitions highlight the interconnectedness of mental health and wellbeing. Mental health is an essential aspect of overall wellbeing, which encompasses physical, emotional, and social dimensions. Positive mental health allows individuals to manage their emotions, develop and maintain meaningful relationships, and make positive contributions to society. It also plays a vital role in enhancing one’s quality of life and achieving a state of overall wellbeing.

The mental health and wellbeing of Chinese international students pursuing their education in Australia has attracted previous attention in the literature. According to Lu et al. (2014), 54% of Chinese-speaking international students in Australia (*n*=144) reported being affected by a high level of psychological distress. In another study by Redfern (2016), Chinese international students in Australia experienced significantly higher levels of anxiety and stress than their Australian counterparts, which was attributed primarily to cultural factors.

More recently, a systematic review conducted by Zhao, Chapman, et al. (2022) identified numerous factors that may affect the mental health of Chinese university students studying in Australia, such as language barriers; social, academic, and financial hardships; the complexities of adapting to the differing educational systems between China and Australia; and students’ underuse of accessible mental health resources. Collectively, these studies indicated that Chinese postgraduate students in Australia often experienced acculturative stressors from language deficiencies, social interaction, academic work, and the different cultural values implied by two educational systems in China and Australia (Chen, 2008; Pan et al., 2008). Most of these studies, however, were conducted among Chinese students who had pursued undergraduate study in Australia. Only a few had focused on, or even included, postgraduate students in Australia.

A small number of studies have been conducted from the interpretivist paradigm with staff members associated with international students. In one example, a qualitative study was conducted by Forbes-Mewett and Sawyer (2016), in which international student support staff, counsellors and academic staff in one Australian university were interviewed. Results suggested that students experienced a significant increase in both the number and severity of mental health problems after arriving in Australia. While this study did not focus directly upon students’ perspectives, results of this kind serve an important purpose in the literature. Most importantly, they are able to provide a more in-depth view of the issues confronted by international students than is possible when taking a standard survey-based approach.

In most previous studies, too, international students of different cultural backgrounds have typically been regarded as a homogenous cohort. This approach is problematic, given that the cultural distances between the host country and the home countries of these students may differ considerably (Zhang, 2013). Cultural distance can be understood as the extent to which the culture in one country differs from another (Raza et al., 2002). The larger the cultural distance between the host country and the home country, the more difficult people are likely to find it to adjust to the host country (Bochner, 2003). According to Hofstede’s “culture-climate map” there is a high cultural distance between Australia and China. Moreover, Chinese culture differs more from Australian culture than from that of some other Western countries, including the UK and the US. On that, whilst we recognize that applying Hofstede’s framework may oversimplify the diversity of Australian culture, this is a well-established model for exploring broad differences across cultures. Accordingly, Chinese students studying in Australia may experience more difficulties in adjusting culturally to their host country than their counterparts studying in other Western countries.

The remainder of this paper, it will be recalled, arose from a larger project on the perspectives of Chinese international students on their studies in Australian universities. It was pointed out already that the dominant issues raised by participants pertained to their mental health and wellbeing. The subsequent focused study that was conducted with the aim of generating theory on their perspectives in that regard is now reported.

All students in the present study were at the end of their first year of enrolment. Participants were all Chinese national students who were born and grew up in mainland China, and who spoke Mandarin Chinese as their first language. At the time the study was undertaken, all were enrolled in either a Master’s or a Doctoral program at an Australian university. While the aim of the study was conceptualized broadly at the outset (i.e. to develop an understanding of the experiences of Chinese students at an Australian university), the themes that emerged from the results focused primarily on risks associated with mental health. The latter overarching theme, therefore, was generated empirically from the data collected.

## Methodology

The researchers’ study gained ethics approval from The University of Western Australia’s Ethics Committee (Approval # RA/4/20/5661). Informed consent was obtained from all participants, and the procedures followed were in accordance with the Australian Research Council National Statement on Ethical Conduct in Human Research. A qualitative design (Clarke et al., 2015) using conversation-style interviews with volunteers who had completed an undergraduate degree in mainland China was conducted. All were attending an Australian university. Some of those were enrolled in a doctoral degree and others in a Master’s level coursework-based or a research-based degree.

The remainder of this section is set out in accordance with the approach used by Khalil and Lee (2018). Overall, the present authors set out to generate theory about potential risks identified in relation to the mental health and wellbeing of students of the “type” studied as revealed in semi-structured interviews. The underlying epistemology stemmed from the interpretivist paradigm (Deetz, 1996). In studies undertaken within this paradigm (Blumer, 1969), there is a focus on “social interaction as the basis for knowledge” (O’Donoghue, 2018, p. 9) and on knowledge being constructed by mutual negotiation. It is also specific to the situation being investigated (Aksan et al., 2009).

The researchers sought volunteers so that no student would feel pressured to be involved in the study. Purposive selection procedures were then used within this group of volunteers, as detailed by Morse (1991) and Barbour (2001). That required selecting those who had particular knowledge and experience, while also ensuring maximum variation (Sandelowski, 1995) within the total pool in order to canvass as wide a range of perspectives as possible. The researcher followed an *aide memoire* based on investigating the following themes: participants’ intentions regarding their studies, the strategies they said they used for realizing those intentions, the significance they said they attached to their intentions and strategies, and the outcomes they said they expected would eventuate from pursuing those.

The above themes were developed based on the interpretive approach by Blackledge and Hunt (2017). To understand everyday activity, we need to understand the *meanings* that people assign to their behaviour. However, the term “meaning” is complex and often undefined, encompassing notions such as aims or intentions, significance, and reasons. These meanings are personal to the individuals involved, constructed from their cultural context rather than being dictated by society. To effectively analyse everyday activity, it is essential to consider the diverse and nuanced meanings that individuals attribute to their actions. Another important element of this framework is that of interaction, in which parties involved attempt to impose their definitions of the situation on each other, using various techniques or strategies to maximize their chances of achieving their own goals. This highlights the dynamic nature of social interactions, in which individuals continuously negotiate and navigate to assert their perspectives and fulfill their objectives.

In accordance with the tenets of interpretivist research (O’Donoghue, 2018), participants were also constantly probed to try to uncover the reasons they gave for what they said. Field notes were initially taken by the researcher conducting the interviews. All interviews were then transcribed verbatim and rechecked for accuracy by the researcher for subsequent analysis.

### Participants

In total, 19 Chinese students studying at an Australian university at the end of their first year of enrolment participated in the study. Among them, nine were males and ten were females; ten were enrolled in Masters’ coursework degrees, six were undertaking PhD studies and three were doing Masters’ coursework degrees combined with research. The average age was 24.74 years, and the length of each student’s stay in Australia ranged from seven months to three years. More information can be found in Table I.

**Table I.** Demographic information on participants.

No.	Name	Age	Sex	Length of Stay in Australia	Degree: Master’s or PhD
1.	Yulan	26	Female	1 year	Master’s
2.	Xiaoming	31	Male	1.5 years	Master’s
3.	Laodong	27	Male	3 Years	Master’s
4.	Gairui	22	Male	1 Year	Master’s
5.	Baoyun	23	Male	1 Year	Master’s
6.	Jie	23	Female	1 Year	Master’s
7.	Bowen	26	Male	2 years	Master’s
8.	Biran	27	Male	9 months	PhD
9.	Wenrou	23	Female	14 months	Master’s
10.	Lishuang	23	Female	7 Months	PhD
11.	Kaka	22	Male	14 Months	Master’s
12.	Dandan	28	Male	1 Year	PhD
13.	Zuozuo	25	Male	7 Months	PhD
14.	Qiumin	26	Female	8 months	PhD
15.	Moming	23	Female	20 months	Master’s
16.	Shilu	23	Female	1 year	Master’s
17.	Jiefang	26	Female	1 Year	PhD
18.	Meili	23	Female	1 Year	Master’s
19.	Lili	23	Female	1 Year	Master’s

### Procedure

Appropriate approvals from The University of Western Australia’s Human Research Committee were obtained. Participants were given an information sheet at the time of the interview, and informed consent was obtained at the interview. The interviews were semi-structured in-depth interviews and were conducted on a one-on-one basis, taking into account during questioning the view that “wording and sequence may be tailored to each individual interviewee and the responses given, with prompts and probes” (Cohen et al., 2018, p. 511).

Interviews were conducted in Mandarin Chinese to ensure that language difficulty did not hamper participants’ expression. Each interview lasted for 45 to 60 minutes, with all interview data collected over a six-week period during 2019.

Two of the researchers are Australian academics and one (the first author) is an academic researcher, who was herself a Chinese international postgraduate student. The interviews were conducted by the latter researcher. That was valuable as she found it easy to communicate with the students and to show empathy with them during the interviews. At the same time, under the guidance of the other two researchers, she engaged in a substantial number of “bracketing” exercises such as writing memos and notes (Tufford & Newman, 2012). Bracketing is a method used in qualitative research to mitigate the potentially deleterious effects of preconceptions that may taint the research process. For example, the first author wrote memos during the entire data collection and analysis process, to document and reflect on her personal thoughts, feelings, and biases. This helped her to acknowledge her preconceptions and separate them from the data analysis process. In addition, after each interview, she undertook a debriefing exercise with her two colleagues. That was to ensure that any bias that might have entered into conversations was ruled out in subsequent interviews.

The following central research question was developed based on the central aim of the initial, broadly-focused study: *What theoretical constructs can be generated regarding Chinese students’ perspectives on their studies at an Australian university, at the end of their first year of study?* On central questions of this kind, O’Donoghue (2018) argued that perspectives can be seen as having four interrelated parts that can be used to generate more specific research “guiding” questions. These parts relate to participants’ “intentions”, “strategies”, “significance”, and “outcomes”. O’Donoghue proposed further that participants can also give *reasons* for what they state in relation to each of these. Adopting the latter approach led to the generation of the four guiding questions detailed in Table II below.

**Table II.** Research guiding questions.

1	What are your intentions regarding your studies for the remainder of your period of study in Australia, and what reasons do you give for having these intentions?
2	What strategies do you have for realizing your intentions regarding your studies for the remainder of your course, and what reasons do you give for having these strategies?
3	What significance do you attach to having these intentions and strategies, and what reasons do you give for having them?
4	What outcomes do you expect will eventuate for you from pursuing your intentions and strategies regarding your studies for the remainder of your course/study, and what reasons do you give for having these outcomes in mind?

Such guiding questions, according to O’Donoghue (2018, p. 37), are not “logical subsets of the general research question”. Rather, they are questions to be drawn upon to generate an initial set of conversation questions aimed at engaging “participants in conversations across a very wide range of areas in order to yield data regarding their perspectives” (p. 37). In this approach, once interview conversations are pursued, there should be no attempt to force answers. Relatedly, unanticipated questions that arise should be pursued. As noted previously, while the starting questions were constructed quite broadly, the nature of the data produced led logically to the generation of themes primarily to do with mental health. That was because issues in that domain were highlighted more than any others by participants.

At the end of each interview, each participant was asked a “clearing-house question” (Minichiello et al., 2008, p. 113) such as “Is there anything that you might not have thought about before that occurred to you during this interview?” or “Is there anything you would like to ask me?” Participants who raised concerns within their interviews for which there were existing university support services were provided with further information on these services following the interview. Interviews were audio-recorded, identifying details were removed, and transcriptions were made and rechecked for accuracy by the researchers.

### Data analysis

Each interview was transcribed soon after it was conducted. Respective transcriptions were then sent to each participant to confirm that these provided an accurate account of what that participant said. The translation process was meticulously executed to ensure the preservation of meaning and the integrity of the research findings. The translations were conducted by the first author, who is also a certified Chinese-English translator. All transcriptions were translated before coding, allowing for seamless integration of the translated data into the analysis. Throughout the translation process, the researcher also consulted with language experts and native speakers of both English and Chinese to maintain the cultural nuances of the original data and ensure the fidelity of the translations. All of these efforts helped to minimize any distortions of meaning and thus helped to maintain the overall integrity of our research findings. The researchers immersed themselves in the data and read and re-read in an iterative process to familiarize themselves with the data, make sense of it, and make initial analytic observations. Data were then analysed using a grounded theory approach. This is a process of conceptual abstraction by assigning general concepts (codes) to singular incidences in data (Vollstedt & Rezat, 2019).

In grounded theory, coding is the process of analysing the data (Walker & Myrick, 2006). According to Saldana (2009), coding is a process with three phases of open, axial and selective coding, which progressed “from codes to categories to theory” (p. 4). Open, axial and selective coding with the data collected results in theory creation, which allows the researchers to construct theoretical meanings, in the form of themes and patterns, at a deeper level (Williams & Moser, 2019). For internal coherence and comprehensiveness, all of the researchers coded the data and generated the initial codes and themes. Interpretation of themes and codes was then conducted by a process of repeat checking against the dataset to ensure an accurate representation of the total body of data generated.

### Trustworthiness of the study

The aim of the study was not to generalize but to “provide a rich, contextualized understanding of some aspect of human experience through the intensive study of particular cases” (Polit & Beck, 2010, p. 1451). More specifically, the aim here was to provide a rich contextualized understanding on the perspectives of Chinese international students on their studies in Australian universities in general. To this end, trustworthiness was addressed by attending to credibility, transferability, dependability and confirmability (Lincoln & Guba, 1985). Quoting original words from participants and member checking were carried out to increase credibility. The transferability and dependability of the study were enhanced through the use of rich descriptions, in addition to explicating in this paper the study’s purpose, participant selection, data collection and data analysis approaches. Field notes were maintained throughout the interviews to enhance the confirmability of the results.

## Results

After the open coding process, specific codes such as “lack of security”, “discrimination”, “feeling lonely”, “feeling homesick”, “increased academic pressure”, etc. were obtained. Then the axial coding resulted in broader categories. For example, “lack of security” and “discrimination” were all categorized into a broader cluster, “perceived safety”. The last phase of selective coding then allowed the researchers to construct theoretical meaning at a deeper level (Williams & Moser, 2019). Three broad themes and nine sub-themes within these were generated based on these analyses.

### Broad theme 1: psychological stress

The first broad theme that emerged related to experiences of psychological stress amongst students. Psychological stress was defined by Lazarus and Folkman (1984, p. 19) as “a particular relationship between the person and the environment that is appraised by the person as taxing or exceeding his or her resources and endangering his or her well-being”. Within this broad theme, three main contributors to increases in psychological stress were identified by students: stress from language barriers; pressure from coping with shifts in pedagogy; and stress caused by changes in lifestyle.

#### Subtheme 1.1: stress from language barriers

Language barriers were seen to introduce pressures in relation to classroom and academic exchange situations. On this, Xiaoming commented: “The situation at the beginning of the study wasn’t very good … my English was not very good … I couldn’t understand the lectures”. Meili also shared her struggles in the study due to language barriers, “the first semester was particularly bad … because of language barriers, it took me about five or six times the time of others to get things done … and I was quite nervous and stressed”.

Meili mentioned too that while lecture recordings helped, not all of her courses provided recordings, which threw her into a feeling of helplessness:

… (for those that have recordings), if I didn’t understand any content of the subject, I could just watch the recording again and again after class, although it was time-consuming … for those without recordings, if I didn’t understand during the class, there was nothing I could do. I didn’t even know how to prepare for the final exams.

Similarly, some PhD students reported difficulties they experienced while communicating with their supervisors. Zuozuo, for example, said: “I couldn’t understand what he (supervisor) said … I feel stressed out”.

Low English proficiency may also introduce pressure on Chinese students by making it difficult for them to make friends with local students. Barriers that the participants cited included being unable to understand jokes and slang in Australian English. On this, Meili shared her own story, saying: “I can’t hang out with local students, because it’s very likely that I won’t understand their jokes. I also can’t keep the conversation going because of this”. She went on to list other specific language barriers as follows:

The English language they use is very local, with slang, and riddles … they speak at a very fast speed, which I can’t keep up with. They also really like jokes, which sometimes I can’t understand … so I just faked laughing when others laughed. This means that it can be stressful to be in social situations here.

Thus, while students like Meili may reach the entry level of language competence required by Australian universities, they are still likely to experience myriad language difficulties in listening and communicating. These difficulties were all cited as stressors that made it more difficult to maintain positive mental health and wellbeing in the first year of study in Australia.

#### Subtheme 1.2: pressure from coping with shifts in pedagogy

Students in the present study cited numerous difficulties they encountered in terms of the pedagogical approaches they were exposed to. In particular, several commented upon the strong emphasis on group work activities in Australian universities. These students cited difficulties in communicating or cooperating with other students from different cultural backgrounds during such activities. On this, Moming claimed that some of her classmates were “very uncooperative” during group work, stating “One [student within my group] rarely participated in our group discussion, and basically contributed nothing when he participated … it’s really hard to communicate with him sometimes”. Xiaoming also shared his experience, “In China, there wasn’t as much group work as here. I encountered some difficulties while doing group work … it is sometimes very difficult to work with people from other cultural backgrounds”.

#### Subtheme 1.3: stress caused by changes in lifestyle

For Chinese students, in particular, their lifestyles in Australia are invariably very different from their lifestyles in China. As Moming shared:

Here I feel that everyone is living far away from the campus. While studying in China, everyone lived on campus in a dormitory, and ate in the cafeteria … there is no cafeteria on campus here like that in China, and we have to learn to cook by ourselves. So that can be stressful at times.

Bowen shared a similar experience. “While studying in a Chinese university, you don’t need to cook yourself, you don’t need to think about rent and utility bills, you don’t need to drive to university because you live in a dormitory … you don’t need to think about various kinds of insurance, and visas and medical examinations”. “But when you study abroad”, he concluded, “you have to deal with all these things on your own”. In addition to dealing with the academic pressures imposed upon them, such shifts will inevitably increase stress levels on these students.

### Broad theme 2: perceived safety

The Chinese students interviewed in this study cited safety as a significant concern while living and studying in Australia. According to the definition given by the World Health Organisation (WHO), safety has two dimensions: an *objective* dimension, “which can be seen as behavioural and environmental factors measured against *external* criteria”, and a *subjective* dimension, which can be variously defined as the individual’s *internal* feelings or perceptions of being safe” (Nilsen et al., 2004, p. 71). Three sub-themes emerged within this general theme, from the interview data. The safety issues mentioned by Chinese students studying in Australia include lack of security, the safety of possessions, and racial discrimination. These three sub-themes represented both “objective” and “subjective” dimensions of safety identified by the WHO.

#### Subtheme 2.1: lack of security

While many residents in Australia cite concerns about increasing the use of tools such as closed-circuit television cameras (CCTV) for privacy reasons, Chinese international students are more accustomed to a higher level of security in their daily lives. This was cited by several students as a safety concern. Baoyun, for example, noted that “security is a very serious issue … there are not as many CCTVs here … I feel like even if someone takes me away and buries me in the ground, no one can ever find me. I don’t feel safe at all”.

Bowen concurred with this view, commenting: “I don’t think it is safe … there is no surveillance video. If someone is missing, he/she is just missing and will never be found”. Moming also expressed concerns about safety, especially at night: “I feel psychologically unsafe. It’s a big city, and it is dark everywhere during the night … , particularly in the evening and night. Even if I go out with my boyfriend at night, I don’t feel safe”.

Students also drew attention to security on the streets. Relatedly, Lili described her experience of being harassed by an offensive teenager. In doing so, she explicated another layer of concern, namely, that “the law here protects Australian teenagers rather than foreign students”.

In elaborating on her view, Lili said that she is afraid that her visa status might be in jeopardy if “I report conflicts that happened to the police”. Bowen, in a similar vein, suggested that international students should live with other students, adding that “as an international student, I think it would be safer to live together with a few other students … after all, you are in a foreign country”. He also expressed concerns about the possibility of aggression from those he encountered in the city at night, commenting that “some of them may ask you for money. If you don’t give it to them, they’ll become violent. Being with a group of people is safer”.

#### Subtheme 2.2: safety of possessions

Xiaoming stated that security is “definitely a problem”, sharing his experience of having his computer stolen:

I left my computer in the living room … and it was stolen … many students had experienced having their things stolen. It is useless to call the police. One of my friends had such an experience … he called the police, but they (the police) didn’t help at all.

Moming revealed her experience of her passport being stolen: “We parked our car in a public parking lot in Northbridge and went to have breakfast. But we came back and found that the car window was broken into pieces … our passports were stolen. This makes us feel very unsafe”.

#### Subtheme 2.3: racial discrimination

Students in the present study reported feeling high levels of discrimination in various ways. Lili, for example, described one unpleasant experience: “I was waiting at the bus stop alone. Suddenly, someone in a car rolled down his window and shouted at me ‘*** Asian’ … I was very scared”. Gairui also shared his experience “We were walking on the street, and suddenly a car was passing by, some people in the car shouted to us ‘Ching-Chong’. This is a very discriminatory name for Chinese people … very insulting”.

Discrimination was also reported to happen on campus. Gairui, for example, shared his experience of being verbally attacked in the residential college in which he was living, “When I introduced myself and said that I came from China, a local boy said to me, ‘You Chinese people really like cheating, don’t you?’”.

Along with more blatant acts of discrimination as mentioned above, some other forms of discrimination were expressed in more subtle forms. Moming commented upon such subtle forms of discrimination in describing her typical shopping experiences: “Salespeople are generally just nicer to the customers of their own race than they are to us”.

### Broad theme 3: social isolation

Relocating to a new country may bring a sense of social isolation for international students. The Australian Institute of Health and Welfare defined social isolation, as “the state of having minimal contact with others” (Australian Government, 2021). While living in the host country, these students no longer have access to familiar cultural norms and social support systems (Chuah & Singh, 2016) and may experience some changes in their identities and concepts of self (Bhugra & Becker, 2005; Jibreel, 2015).

#### Subtheme 3.1: lacking a sense of belonging

In the study conducted by the present authors, students frequently mentioned that they feel a lack of sense of belonging while studying in and living in Australia. For example, Gairui said that he felt a lack of sense of belonging because there is no homogenous class group in Australian universities (In this context, class refers to “a group of students who are taught together at school, college, or university” https://dictionary.cambridge.org/dictionary/english/class):

While studying in China, everyone had a class, and we were all together with a class of classmates … we were closer and had classes together … here there is no class to which we belong, and relationships among students are further. We got to know new students from each course but lost contact when the course ended…

Another participant, Laodong, claimed that he does not feel he belongs here, and that he misses the familiarity with others when living in China: “I miss the familiarity of living in China … people are all Chinese … there are no language or communication barriers. Communication there is much easier”.

#### Subtheme 3.2: lacking close personal connections

Xiaoming gave voice to the lack of close personal connections, stating that “Most students are unfamiliar with their classmates”. Moming concurred as follows: “You basically finish class and just leave. Everyone is busy”. Yulan shared a similar experience: “I have no very good friends here … most of the time, I do everything on my own”. Shilu also said, “I have some acquaintances, but not friends, not to mention close friends whom you could open your heart to”. Students mentioned too that mature age, related interests, language and cultural barriers, and stereotypes may all prevent them from establishing close connections with others. For example, Meili stated that “it is difficult to continue our communication due to the lack of common ground and different cultural backgrounds”.

#### Subtheme 3.3: feelings of loneliness and homesickness

In the study reported here, students said that they experience homesickness and loneliness during their stay in Australia, especially at the beginning of their study period. Bowen recalled his feelings on when he first arrived, stating: “I really wanted to go back to China because there are not diversified takeaways delivered here, there are not many entertainment facilities or nightlife”. Others had that their loneliness comes mainly from having no friends. Jie said, “It was really a feeling of loneliness, especially last semester … I didn’t find a good way to make friends. I was very lonely”.

She further added that her family and friends in China could not provide any support from a distance because they could not understand why she felt lonely:

My mother couldn’t understand why I feel lonely here because I am already living in a residential college, and I am supposed to have a lot of friends there. My friends in China couldn’t help me either, because they are not living here, and don’t know the situation here.

Jiefang shared a similar experience: “I had no one to talk to. When I got home every day, I was alone. My housemate always stayed in her room and rarely came out. I felt very sad and lonely at that time”.

Bowen added in like manner “I felt quite lonely, it’s very uncomfortable when you have no one to talk to”.

Despite the loneliness experienced by students, most say that they tried hard to adjust to these circumstances. Others, however, ended up giving up. For example, Yulan had maladjustment when she first arrived in Australia, and ultimately returned to China after finishing a bridging course: “I returned to China after the bridging course because I felt very lonely. I had no friends here, and life here was boring, I didn’t know what to do every day especially when I had no friends”.

Shilu, in like manner, voiced that many “traditional Chinese festivals like Mid-Autumn Festival” make her feel particularly homesick.

## Discussion

It has been clear for some time that international students may be vulnerable to mental health issues following relocation to Australia. Chinese international students are no exception in that regard. Three categories of risk factors that potentially affect Chinese students’ mental health have been identified in the study. The following discussion will be divided into two parts. The potential impact of the three categories of risk factors on Chinese international students’ mental health is first discussed. Recommendations for practice are then made based on the findings of the study.

### Three categories of risk factors for Chinese international student

The first broad theme that emerged in this study related to psychological stress. The factors that emerged through the interviews are consistent with findings from previous studies conducted with international students. These prior findings have suggested that language barriers can cause stress in relation to academics and social life among international students (Ching et al., 2017; Gatwiri, 2015; Wu et al., 2015). Similarly, in the present study, language barriers and low English proficiency were both seen to have a negative influence not only on academic performance, but also, on the social lives that mainland Chinese students were afforded whilst studying in Australia.

Experiencing a very different pedagogical approach to the ones they are used to experiencing in their home countries may also bring stress to Chinese students. The pedagogy in China has been characterized as typically “teacher-centred”, in which students generally do not interact with one another much in class or challenge the opinions of their teachers (Newsome & Cooper, 2017; Phillips et al., 2002). As a result, experiencing the kinds of pedagogical approaches that are used in Australian education, which tend to encourage critical thinking (Egege & Kutieleh, 2004), group work (Riebe et al., 2017) and self-directed learning (Stewart, 2007), can be a stressful experience. These pedagogical differences can be especially challenging for postgraduate students who have already experienced the Chinese educational system during their undergraduate studies, in a similar way to the participants in the present study. They may find it difficult to adapt to the new academic expectations, engage in class discussions, and navigate the more independent nature of graduate-level coursework or research.

The lifestyle changes that international students encounter upon moving to another country can also be stressful. On that, Newsome and Cooper (2016) explained that people who first encounter alien cultures in the early stages of adulthood often experience a sense of dislocation when adjusting to “an unfamiliar culture, economy, education, family, government and society without the benefit of the many years of gradual socialization that most indigenous members of the host culture have experienced” (p. 195). International students, while adapting to a new living and educational environment, must also navigate the unfamiliar culture, economy, family dynamics, and society of their host country. They face these challenges without the benefit of the years-long socialization process that most native students of the host culture and other international students who are from a country with similar culture may have experienced.

On confronting such changes, individuals are likely to experience varying levels of stress (Wisse & Sleebos, 2016), which can pose a threat to mental health at excessive levels or in prolonged form. Chronic stress has been found to increase both the risk of developing depression and anxiety (MQ Mental Health, 2018), and also alcohol and drug use, both of which may cause further problems for the individual in the longer term (e.g. Robinson, 2017; Sinha, 2008).

The second broad theme that emerged is perceived safety, which has long been recognized as a significant factor in mental health and functioning (see Booth et al., 2012). In a study involving 95,712 students, Etopio et al. (2019) reported that perceived safety was particularly important as a moderator of mental health in university-level students. Huang et al. (2020) reported similar results in a study on Chinese students studying in Australia. Similarly, students in the present study reported that feeling “unsafe” impacted their overall sense of wellbeing. Increased racial discrimination can be a source of such feelings, and has been associated with poor mental health outcomes (Cheah et al., 2020; Ferdinand et al., 2015). Noh et al. (2007), for example, indicated that perceived racial discrimination was associated with both the erosion of positive affect and depressive symptoms.

The third broad theme that emerged in this study was that of social isolation, which has been reported as potentially harmful to one’s mental and physical health (Australian Government, 2021). The subthemes that emerged under social isolation included lacking a sense of belonging, lacking close personal connections, and feelings of loneliness and homesickness. Sense of belonging has been defined as “the experience of personal involvement in a system or environment so that people feel themselves to be an integral part of that system or environment” (Hagerty et al., 1992, p. 173).

For international students to achieve a sense of belonging, they need to become involved in their new academic and social environments, which can include participating in class discussions, joining clubs or organizations, engaging with local communities, and forming meaningful connections with peers, faculty, and staff. However, achieving this sense of belonging can be challenging for international students for various reasons. International students’ values and norms are likely to be different from their mainstream student communities (Van Gijn-Grosvenor & Huisman, 2020), which may in turn contribute to a reduced sense of belonging (Hoffman et al., 2002). Previous research has indicated that lack a sense of belonging is associated with “more frequent doctor visits, and more frequent reports of loneliness, depression and suicidal thinking” (see Van Gijn-Grosvenor & Huisman, 2020 for a review, p. 378).

International students may experience difficulties in making friends while living and studying in a host country, not to mention missing close friends from their home countries. According to a report in the *Journal of International and Intercultural Communication* (Niekerk, 2012), in 2012, 40% of international students in the United States reported having no close friends amongst their academic peer groups. Students included in the present authors’ study also expressed that they feel it is difficult to make close friends in Australia regardless of nationalities. One possible reason for the latter situation, according to students, is that the nature of relationships that exist among students in the two countries is very different. In Chinese universities, they held, students are normally very close in their personal relationships, with classmates usually spending a lot of time together both in class and when participating in collective activities throughout their four-year undergraduate studies or their two/three-year Master’s studies. In general, however, most students said they feel that in Australia, while students tend to greet one another, they live their lives separately without spending much time together. A consequence, they argue, is that it is difficult for Chinese students to make friends while studying in Australia.

Loneliness and homesickness constitute another challenge faced by international students. On those, Banjong (2015) found that loneliness and homesickness can have a negative influence on international students’ academic performance as well as on their mental health by causing stress and depression. Such negative results, they go on to say, may also further prevent students from taking initiatives to socialize with others.

Lacking a sense of belonging, lacking close personal connections, loneliness and homesickness all may provoke a grief reaction (Eisenbruch, 1990, 1991), which, if prolonged, can precipitate psychiatric symptoms (Bhugra & Becker, 2005). That is instructive in relationship to the study conducted by the present authors, as Chinese students experienced lacking a sense of belongingness and lacking close personal connection. Sometimes, they said, they also felt lonely and homesick. All of these experiences could lead to grief, which in turn can cause distress that will, ultimately, lead one to have negative mental health issues.

The study reported in this paper highlighted a number of potential risk factors with respect to the mental health of Chinese international students in Australia. Negative mental health indicators (including fear, stress, anxiety, depression, decreased wellbeing and social problems) can also have reciprocal effects on one another. For example, social problems can increase anxiety in such students, which can, in turn, lead to further social problems and depression (National Collaborating Centre for Mental Health, UK, 2013), which can then cause the person to withdraw, adding to further social problems. Thus, Chinese international students studying in Australia may confront “downward spiral effects” which have significant long-term effects on their mental and physical health. As a result, it is important that these students’ experiences are monitored from the outset, so that the potential for such effects is reduced.

Research of the kind presented in this paper may help institutions to identify ways in which such risks can be reduced through institutional policies, practices and processes. For example, further work could be done to enhance the experiences of Chinese students through pre-arrival induction programs, such as mentoring programs or buddy systems (Menzies et al., 2015; Pearce, 2012). Social media platforms that are commonly used in Australia by college students such as Facebook, Instagram, Google Scholar and YouTube may be harnessed for this purpose. By making the experiences of Chinese students more positive, the use of such strategies could be an important first step in the recovery of the Australian higher education system from the devastating impact of the COVID pandemic.

In addition to increased efforts and strategies initiated by universities or institutions, there are strategies that students themselves can adopt to proactively maintain their mental health. First, students can build their social networks by joining clubs or other interest groups to make friends (The Times Higher Education, 2022). Joining such groups would also increase students’ sense of belongingness and reduce homesickness and loneliness. At the same time, students can also keep learning English and become more familiar with other students from different cultures, which has the potential to increase their confidence while communicating with others as well as increase their sense of belongingness.

Second, students should also be aware of the early signs of poor mental health so that they are well-positioned to seek support in a timely manner. These signs can include sleep disturbances (Nutt et al., 2008), changed appetite (Simmons et al., 2016) and chronic pain (Sheng et al., 2017). Loss of interest in work or study may also be a sign of impending mental health problems (Guajardo et al., 2011). Students may be equipped with relevant knowledge about these signs through workshops provided by universities. Further, once students recognize any signs of mental health problems, they should ensure that they are aware of resources they can access for help and what support and resources are available to them. This may include free on-campus counselling services provided by universities (The University of Melbourne, 2022; The University of Western Australia, 2022) as well as mental health support and services provided by other organizations (e.g. Beyondblue, 2022; Butterfly, 2022).

## Conclusion

This paper reported a study on the experiences and challenges of Chinese international students at an Australian university by interviewing 19 such students and analysing data using grounded theory. Three broad themes on risks associated with mental health were generated: psychological stress (which was linked to language barriers, shifts in pedagogy, and changes in lifestyle); perceived safety (which was linked to lack of security, safety and perceived racial discrimination); and social isolation (linked to reduced sense of belonging; lacking close personal connections; and feelings of loneliness and homesickness). Results suggest that a tripartite model of interactive risk factors (psychological stress, perceived safety and social isolation) may be appropriate for examining the mental health of international students. Finally, strategies for universities and students were put forward to maintain and support international students’ adjustment during their study periods.

Importantly, all studies of the kind presented here are necessarily bound by the contexts in which they were conducted. Thus, they are not generalizable in the positivist sense. Their value lies in helping others to clarify their own perspectives and also to offer suggestions that may be put to the test of practice to determine whether they work for others in similar circumstances. Finally, we hope that others will be encouraged to adopt the research approach we detailed to engage in a greater range of studies of the type reported here. This will ensure that initiatives to assist Chinese international students are informed not only by results from “outsider” investigation, but also, by those who are “insider” in nature.
